# Synaptic Effects of Dopamine Breakdown and Their Relation to Schizophrenia-Linked Working Memory Deficits

**DOI:** 10.3389/fnsyn.2018.00016

**Published:** 2018-06-12

**Authors:** Andrew D. Bolton, Martha Constantine-Paton

**Affiliations:** ^1^Center for Brain Science, Harvard University, Cambridge, MA, United States; ^2^McGovern Institute for Brain Research, Massachusetts Institute of Technology, Cambridge, MA, United States

**Keywords:** NMDA, dopamine, working memory, schizophrenia, homocysteine, persistent activity

## Abstract

Working memory is the ability to hold information “online” over a time delay in order to perform a task. This kind of memory is encoded in the brain by persistent neural activity that outlasts the presentation of a stimulus. Patients with schizophrenia perform poorly in working memory tasks that require the brief memory of a target location in space. This deficit indicates that persistent neural activity related to spatial locations may be impaired in the disease. At the circuit level, many studies have shown that NMDA receptors and the dopamine system are involved in both schizophrenia pathology and working memory-related persistent activity. In this Hypothesis and Theory article, we examine the possible connection between NMDA receptors, the dopamine system, and schizophrenia-linked working memory deficits. In particular, we focus on the dopamine breakdown product homocysteine (HCY), which is consistently elevated in schizophrenia patients. Our previous studies have shown that HCY strongly reduces the desensitization of NMDA currents. Here, we show that HCY likely affects NMDA receptors in brain regions that support working memory; this is because these areas favor dopamine breakdown over transport to clear dopamine from synapses. Finally, within the context of two NMDA-based computational models of working memory, we suggest a mechanism by which HCY could give rise to the working memory deficits observed in schizophrenia patients.

## Introduction

The purpose of this Hypothesis and Theory article is to lay groundwork for linking two findings that are typically observed in subjects with schizophrenia: spatial working memory deficits and elevated homocysteine (HCY) levels. We briefly explain these two findings, simultaneously illustrating their possible relationship within a framework of dopaminergic physiology. We next present a theory within the context of computational models of persistent neural activity through which HCY may affect neural circuits that mediate spatial working memory. Finally, we suggest future experiments based on our theory.

## Two Seemingly Unrelated Deficits Observed in Schizophrenia

**Figure 1:**
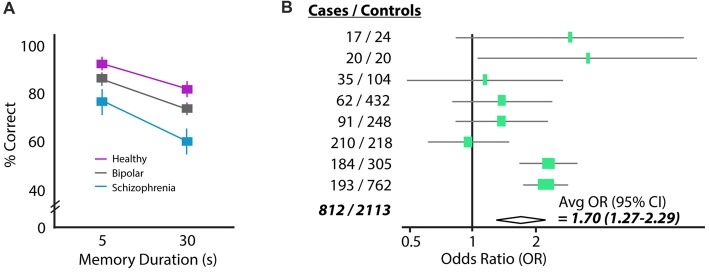
Two common findings in schizophrenia: working memory deficits and elevated homocysteine (HCY). **(A)** Performance of a schizophrenia, bipolar and control cohort on a delayed saccade task. Subjects must remember the location of a target over a brief delay (5 or 30 s) and move their eyes to the remembered location (reprinted with permission from Park and Holzman, 1992). Schizophrenia patients perform significantly worse than either bipolar or control cohorts. **(B)** The amino acid HCY is consistently elevated in the blood of schizophrenia patients. Shown are the odds ratios and 95% confidence intervals for having schizophrenia given a 5 μM increase in plasma total HCY. Eight total case-control studies were analyzed in this meta-analysis, showing the total increase in schizophrenia odds to be 70% given a 5 μM increase in HCY (reprinted with permission from Muntjewerff et al., 2006).

Schizophrenia patients frequently show deficits in spatial working memory. When a subject with schizophrenia is asked to remember a spatial location over a short delay of 5 or 30 s, they perform significantly worse than both healthy controls and cohorts with bipolar disorder (Figure 1A). The errors schizophrenia patients make in this task tend to be perseverative, meaning that their incorrect answers are correct answers from previous trials (Park and Holzman, 1992). Moreover, the incorrect answers given by patients are provided with high confidence, suggesting there is a “false” memory trace in the brain and not simply an inability to create memory traces (Mayer and Park, 2012). This spatial working memory deficit has been observed in many independent studies and is considered an “endophenotype” of schizophrenia because it is also observed in patients’ healthy first-degree relatives (Park et al., 1995; Cannon et al., 2000; Gottesman and Gould, 2003; Pirkola et al., 2005; Mayer and Park, 2012). These results suggest that there may be a genetic contribution to working memory failure, which resembles the partial genetic basis of schizophrenia itself (e.g., Torrey et al., 1994).

The perseverative feature of answers during working memory tasks appears to reflect a general tendency of schizophrenia patients to perseverate in other tasks. For example, during the Wisconsin Card Sorting Test, schizophrenia patients have repeatedly shown to hold on to previously correct sorting rules even when their currently implemented sorting scheme is incorrect (for review see Crider, 1997). We and others (Goldman-Rakic, 1999) suggest that these deficits stem from a defect in persistent neural activity that encodes remembered locations and sorting schemes. This idea will be addressed in more physiological detail in later sections.

A seemingly unrelated characteristic of schizophrenia patients is the presence of high levels of the amino acid HCY in the blood (e.g., Regland et al., 1995; Levine et al., 2002; Goff et al., 2004). Strikingly, a recent meta-analysis of the linkage between high HCY and schizophrenia suggests that humans with a 5 μmol/L higher than average concentration of HCY in their blood are 70% more likely to have schizophrenia (Muntjewerff et al., 2006; Figure 1B). Significantly elevated HCY levels have also been observed in the cerebrospinal fluid of a large cohort of schizophrenia patients (Regland, 2005; *p* < 0.04, *n* = 36).

High levels of HCY are also observed in other disorders marked by cognitive deficits (e.g., hyperhomocysteinemia, homocysteinuria, fibromyalgia); however, the mechanism by which HCY is related to cognitive deficits and schizophrenia is unknown. Granted, high HCY levels may be an epiphenomenon of schizophrenia: heightened HCY may not induce symptoms but could instead be a byproduct of an unknown dysregulated process that occurs more often in patients. If this were the case, however, it would be surprising that nutritional strategies used to reduce levels of homocysteine have proven to reduce schizophrenia symptoms and improve performance of schizophrenia patients on the Wisconsin Card Sorting Test (Levine et al., 2006).

## HCY Arises After Dopamine Release in Specific Brain Regions

**Figure 2:**
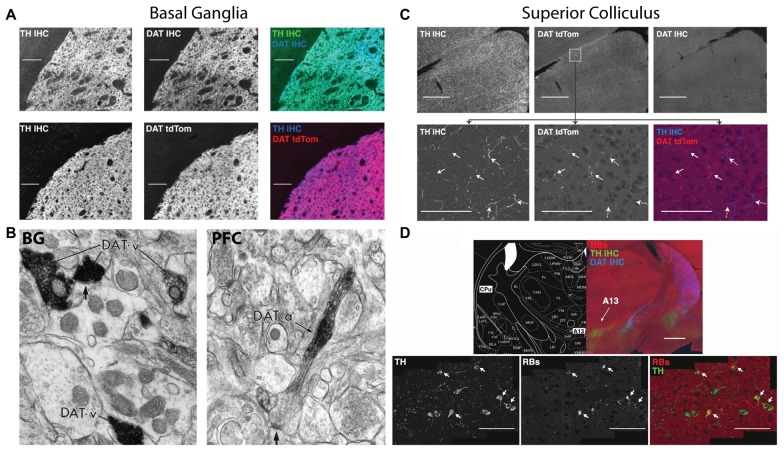
Dopamine transporter (DAT) expression varies across the brain in terms of expression and localization on dopaminergic axons. Immunohistochemistry for tyrosine hydroxylase (TH IHC), the enzyme required for making dopamine, was performed as a double stain with DAT or on a DAT-tdTomato transgenic mouse. In the basal ganglia (BG), DAT and TH strongly overlap in terminals originating from the midbrain dopaminergic cell groups. The DAT in these BG terminals clusters in presynaptic terminals that release dopamine (**B**, left, reprinted with permission from Sesack et al., 1998), indicating that dopamine reuptake is the main mode of action after dopamine release in the BG. The superior colliculus (SC; **C**), on the other hand, receives TH+ axons that are DAT negative. SC axons in the DAT-tdTomato mouse that stain positive for DAT do not actually contain TH. Dopamine axons in the SC arrive from the A13 cell group **(D)**, which is labeled by retrograde injections from the SC (Retrobeads = RB). A13 is a dopaminergic cell group in the zona incerta of the diencephalon that does not express DAT (**D**, top). A subset of TH+ axons in the SC are noradrenergic from the locus coerelus (RBs also found in the LC: data not shown here, but shown in Bolton et al., 2015). Also note that the DAT+ axons coming from the midbrain and terminating in the BG in **(A)** can be seen traversing the diencephalon in **(D)**. Therefore, the two modes of dopamine clearance (DAT+ and DAT−) can both be seen in this figure.

HCY is a breakdown product of dopamine, providing a possible inroad for HCY to affect neurons and synapses and, in turn, induce cognitive deficits. However, there is an underappreciated diversity with regards to the location and frequency by which dopamine breakdown actually occurs in the brain. In fact, dopamine reuptake into the presynaptic terminal by the high-efficiency uptake enzyme Dopamine Transporter (DAT, aka Slc6A3) occurs widely in the brain. Indeed, most dopamine cell groups, including the midbrain ventral tegmental area (VTA) and substantia nigra (SN), express DAT and recycle dopamine for repeated use in their axons’ target zones (Ciliax et al., 1995). In the highly dopamine-innervated basal ganglia (BG), for example, transgenic DAT-tdTomato mice and a specific DAT antibody completely overlap with tyrosine hydroxylase (TH) expressing dopamine terminals emanating from the midbrain (Figure 2A; Tritsch et al., 2012; Bolton et al., 2015). Electron microscopy studies on the BG using immunogold show that these dopamine terminals contain extremely dense packing of DAT at the presynaptic terminal (Figure 2B; Sesack et al., 1998). This high DAT expression and density at the synapse suggests that dopamine recycling is the main mode of dopamine clearance in the BG, which is supported by studies showing a very low ratio of dopamine breakdown products to total dopamine in the BG (1:23 ratio of 3-Methoxytyramine (3-MT):dopamine, Weller et al., 1987).

Despite the prevalence of dopamine reuptake, many brain regions instead utilize dopamine breakdown. At this opposite end of the spectrum is the midbrain superior colliculus (SC), also known as the optic tectum. The SC expresses a striking mosaic pattern of dopamine receptors, with D1 receptors localized to its superficial visual layers and D2 receptors enriched in its multimodal motor layers (Bolton et al., 2015). The dopamine terminals that activate these receptors originate from the A13 cell group and do not express DAT (Figures 2C,D; Bolton et al., 2015). Instead, dopamine breakdown via the enzyme Catechol-O-MethylTranserase (COMT) is likely the main mode of action in the SC, which is suggested by the lack of DAT, the particularly high levels of COMT RNA in the SC, the high level of COMT activity in SC explants, and the ~1:1 ratio of COMT-generated breakdown products to dopamine in the region (Bigl et al., 1974; Weller et al., 1987).

The prefrontal cortex (PFC) appears to be a hybrid of the SC and BG in terms of DAT and COMT expression. COMT activity and dopamine levels are similar between frontal cortex and the SC (Bigl et al., 1974; Versteeg et al., 1976), suggesting a similar level of dopamine breakdown. Nonetheless, electron microscopy studies show that DAT is expressed in dopamine axons in frontal cortex, but DAT proteins are not localized to the synapse (Figure 2B; Sesack et al., 1998). Instead, DAT is located away from the synapse on the axon shaft, suggesting that dopamine is not immediately reuptaken into the presynaptic terminal as in the BG. Overall, it appears that DAT and COMT cooperate in this region to clear and metabolize dopamine respectively; the shaft location of DAT indicates that it collects dopamine that has significantly diffused away from synapses.

This continuum of DAT expression at the synapse, ranging from no DAT whatsoever to dense presynaptic DAT, begs the question of why there are multiple means of dopamine clearance at synapses. Why in some brain regions is dopamine recycled while in others it is broken down? We surmise that regions that favor dopamine breakdown via COMT over presynaptic recycling by DAT may utilize COMT-generated breakdown products for ongoing synaptic function.

## How do COMT-Generated Byproducts Affect Synapses?

Although COMT generates multiple breakdown products that are known to affect receptors in the brain (e.g., adenosine, 3-MT, homovanillic acid (HVA)), we focus here on HCY due to its association with schizophrenia.

**Figure 3:**
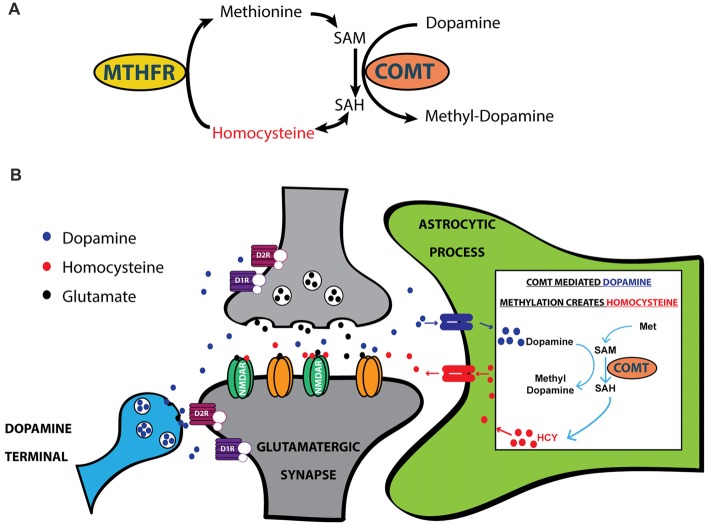
HCY arises after dopamine breakdown by Catechol-O-Methyltransferase (COMT). **(A)** HCY is generated when COMT methylates catecholamine neurotransmitters, including dopamine, to initiate the breakdown process. Free HCY is then remethylated for future catecholamine methylations via a folate-dependent process. The folate derivatives required for HCY methylation are produced by the enzyme Methyl-Tetrahydrofolate Reductase (MTHFR). **(B)** The production of HCY has been shown to occur after dopamine methylation in astrocytes (Huang et al., 2005). Astrocytes then release HCY to the extracellular space, where free HCY can interact with receptors on glutamatergic synapses. SAM, S-adenosyl-methionine; SAH, S-adenosyl-homocysteine.

HCY arises in the brain every time COMT inactivates dopamine (Tunbridge et al., 2008; Figures 3A,B); this occurs when COMT places a methyl group from methionine onto a dopamine molecule (Figures 3A,B). Although there is considerable debate over the cellular and subcellular location of COMT (Schott et al., 2010; Chen et al., 2011), live imaging of COMT immunocytochemistry in cortical cultures suggests an intracellular location of COMT in both neurons and glia (Schott et al., 2010). HCY appears to only be released into the extracellular space if COMT methylates dopamine in *astrocytes*, as revealed by elegant culture experiments in Huang et al. (2005). After astrocytic release, HCY is uptaken into neurons and re-methylated via a folate-mediated pathway that requires the enzyme Methyl-Tetrahydrofolate Reductase (MTHFR; Huang et al., 2005; Tunbridge et al., 2008). In sum, the mode of action in the brain that allows HCY to interact with synaptic receptors begins with dopamine being uptaken into astrocytes (possibly via PMAT: see Schott et al., 2010; Bolton et al., 2015). Dopamine is then methylated by COMT inside the astrocyte, generating HCY that is released back into the extracellular environment.

The synaptic concentration HCY reaches after release from astrocytes is unknown. CSF levels of HCY in schizophrenia can reach ~1 μM but deducing synaptic levels from CSF concentrations is unreasonable considering that dopamine reaches 1.6 mM at synapses after release, but is only at 40 nM in CSF (Gjerris et al., 1987; Regland, 2005). Moreover, since significant HCY production is only expected to occur in the subset of dopamine termination zones that favor breakdown, estimating synaptic concentrations in these specific regions from global CSF seems even more unreliable and warrants further investigation. We hypothesize that if HCY release is confined to the small volume of a glutamatergic synapse, HCY levels could reach similar concentrations to synaptic dopamine (~1 mM) since each inactivated dopamine molecule should create one HCY, and nearly all of the HCY produced in astrocytes is eventually exported out of the cell (Garris et al., 1994; Huang et al., 2005).

**Figure 4:**
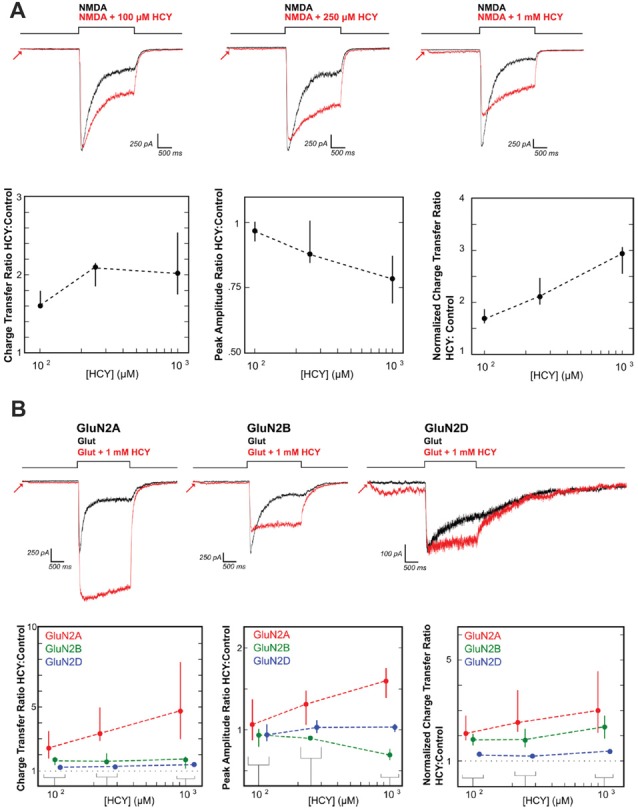
HCY reduces NMDA receptor desensitization. **(A)** Voltage clamped neurons were maintained in 200 nM glycine. NMDA (100 μM) was applied for 2 s both with HCY and without HCY on the same neuron. Increasing HCY concentrations decreased peak NMDAR current amplitude and reduced desensitization dose dependently. Notice that HCY still strongly reduced desensitization at one tenth of its maximal concentration (100 μM = 50 μM active L-HCY isomer; for [HCY] = 100 μM, 250 μM, 1 mM, *N* = 7, 5, 10). **(B)** HCY reduced desensitization of all three NMDAR subtypes transfected into HEK cells to make homogenous GluN2 NMDAR populations. The amplitude of GluN2A responses to glutamate was enhanced by the presence of HCY in a dose dependent manner. GluN2B, similar to NMDARs in young cultured neurons, showed dose dependent amplitude reduction by HCY. GluN2D showed no change in peak amplitude at any HCY concentration [N for each [HCY] (100 μM = 50 μM L-HCY isomer, 250 μM = 125 μM L-HCY isomer, 1 mM = 500 μM L-HCY isomer): GluN2A *N* = 4, 5, 17; GluN2B *N* = 5, 5, 17 GluN2D *N* = 6, 3, 8]. Reprinted from Bolton et al. (2013).

Until recently, the synaptic effects of HCY in the extracellular space after dopamine breakdown were unclear. One initial hint came from studies showing that HCY binds the NMDA receptor and modulates NMDAR-dependent long-term potentiation (Lipton et al., 1997; Christie et al., 2005, 2009). We followed up on these studies using patch clamp electrophysiology in cultured neurons, HEK cells transfected with NMDAR subunits, and neurons in brain slices stimulated with caged glutamate. HCY was shown to severely alter the activation and desensitization kinetics of NMDARs depending on the GluN2 subtype composition of the receptor (Figure 4; Bolton et al., 2013). Specifically, at high concentrations (1 mM = 500 μM active L-isomer), HCY attenuates peak amplitude of GluN2B-receptor currents while augmenting the peak amplitude of GluN2A-receptor currents (Figure 4B). Critical to the theory presented in this article, however, is the fact that regardless of the receptor subtype, HCY strongly reduces desensitization of NMDARs at low concentrations resembling the NMDAR-effective dose of glutamate (100 μM = 50 μM active L-isomer, Figure 4A).

The desensitization reducing role of HCY is very similar to that already described for glycine, d-serine and spermine (Mayer et al., 1989; Lerma, 1992). Glycine, an ambient neurotransmitter, acts as a co-agonist of the NMDAR, meaning that the receptor will not open without glutamate and glycine co-binding. However, the role of glycine is complex in that heightening the concentration beyond the level required for activation continually reduces desensitization of the NMDAR response to prolonged agonist exposure (Mayer et al., 1989). HCY, in fact, likely binds to the glycine site of the NMDAR to enact its desensitization-reducing function: glycine saturation of the NMDAR occludes the desensitization effects of HCY, and DCKA, a specific glycine-site inhibitor, reduces the desensitization preventing effects of HCY (Bolton et al., 2013). Critically, the glycine site is not believed to be saturated by the pool of all glycine-site activators in the brain (e.g., glycine, d-serine; Attwell et al., 1993; Bergeron et al., 1998; Roux and Supplisson, 2000; Chen et al., 2003; Martina et al., 2003; Bolton et al., 2013). We therefore suggest that a previously unappreciated consequence of COMT-mediated synaptic dopamine breakdown is the acute reduction of glycine-dependent NMDAR desensitization by HCY.

## Desensitization of NMDARs and Synaptic Function

**Figure 5:**
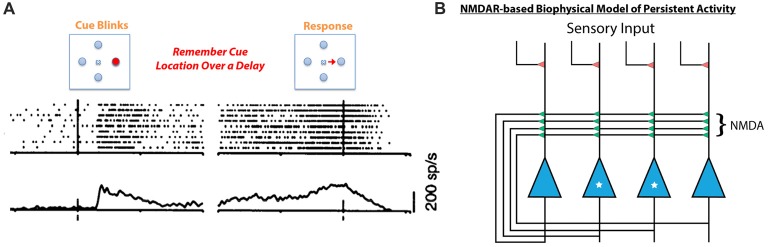
Persistent neural activity mediating working memory is thought to be dependent on NMDA receptor containing feedback loops. **(A)** When a monkey is tasked with remembering a spatial location in the short term, neurons in the SC respond to the initial presentation of the stimulus but remain active after the target has disappeared. This is also consistently seen in the prefrontal cortex (PFC) during working memory tasks (for review see Goldman-Rakic, 1996). This type of persistent neural activity, considered to be the basis for working memory, is mediated by NMDA receptor containing feedback circuits (**B**, Lisman et al., 1998; Seung et al., 2000). In this schema, the two starred neurons’ receptive field is the target to be remembered, while the two un-starred neurons are responsive to other spatial locations. When the starred cells are activated by initial sensory input, they depolarize and relieve the magnesium block in their feedback NMDA receptors. The unstarred cells’ NMDA receptors are still plugged with magnesium. Therefore, the starred cells can continue to fire by driving their own NMDA receptors, sustaining persistent activity over a short delay due to the long-decaying NMDA receptors’ ability to integrate repetitive excitatory currents. This model does not account for the fact that repeated stimulation of NMDA receptors induces desensitization (see Figure 4), which would prevent feedback circuits such as this one from sustaining persistent activity.

Given that HCY strongly affects the desensitization of NMDAR currents, it is critical to explore what role desensitization may play in the brain. Desensitization of glutamate receptors is thought to shape synaptic responses to prolonged input and prevent calcium-induced toxicity during long-term agonist exposure (Lukasiewicz et al., 1995; Stys et al., 2012). Continuous bathing of receptors in agonist likely occurs in the brain when sustained high frequency firing of a presynaptic neuron induces repeated release of agonist into a synaptic cleft. This type of high-frequency persistent firing for seconds-long periods occurs when animals engage in working memory tasks (see Goldman-Rakic, 1996). In fact, persistent excitatory activity is a fundamental form of neural dynamics across the animal kingdom (see Major and Tank, 2004) and is thought to be the neural basis of working memory. Persistent activity, in particular, is thought to mediate memories of spatial locations during the spatial working memory task that schizophrenia patients tend to fail. In this task, a target in a specific region of space is flashed for a moment and the subject must remember the target location over a brief delay (Figure 5A). When monkeys perform the task, neurons in the dorsolateral prefrontal cortex (DL-PFC) and SC that encode the remembered location remain active at high-frequency (~40 Hz) over the entire delay period (Munoz and Wurtz, 1995; Goldman-Rakic, 1996; Figure 5A).

The neural architecture that mediates this type of persistent activity is an excitatory feedback loop whereby the spiking of neurons activated by a stimulus is sustained by recurrent collaterals in the absence of further input (Figure 5B, Lisman et al., 1998; Seung et al., 2000; Wang, 2001). Critically, recurrent excitatory loops created by collaterals exist both in the DL-PFC and in the SC, and their activity is mediated by NMDARs (Pettit et al., 1999; Saito and Isa, 2003; Wang et al., 2013). In fact, computational models of persistent activity related to working memory posit that the long time constant of decay (Seung et al., 2000) and magnesium block (Lisman et al., 1998) of NMDARs make them ideal candidates to mediate persistent activity (Figure 5B, see Figure 5 legend for mechanism). Moreover, experimental work on behaving monkeys has shown that persistent activity related to spatial working memory is blockable by specific GluN2B and GluN2A NMDAR antagonists (Wang et al., 2013). Further work on rodents has shown that rats cannot perform spatial working memory tasks if dosed with NMDAR antagonists (Aultman and Moghaddam, 2001). It is therefore likely that NMDARs play a critical role in the persistently active neural circuits mediating spatial working memory, the precise task that schizophrenia patients fail. Furthermore, intact dopamine signaling through D1 type dopamine receptors appears to be important for maintaining persistent activity in prefrontal cortical networks during working memory tasks (Sawaguchi and Goldman-Rakic, 1994; Goldman-Rakic, 1996), while blocking D1-type receptors in rodents impairs spatial working memory over seconds long delays (Aultman and Moghaddam, 2001). This means that dopamine signaling into persistently active ensembles occurs during working memory tasks, and that HCY production at these ensembles is a likely consequence.

One caveat of models and theories of persistent activity is that they ignore the severe desensitization of NMDARs that occurs within hundreds of milliseconds of agonist exposure. Most theoretical models of persistent activity rely on the continued fidelity of constant NMDAR currents to drive persistently active cells; however, NMDARs desensitize up to 90% after 1 s of agonist exposure (see Figure 4 black control traces). This strong, HCY-blockable desensitization is a feature of both GluN2A and GluN2B type receptors, which are present in cortex and SC, and are required for persistent activity during WM (van Zundert et al., 2004; Wang et al., 2013). The fact that some crucial neural modes like persistent activity require continued NMDAR fidelity makes it interesting that the brain possesses mechanisms that can overcome fast desensitization of NMDARs: namely, the ability to place NMDAR desensitization reducing molecules like glycine, d-serine, or HCY at synapses. We therefore posit that breakdown of dopamine places HCY at synapses to reduce NMDAR desensitization during high-frequency firing. This is supported by the aforesaid facts that COMT is the main means of dopamine clearance in the areas that support high-frequency persistent activity: the SC and PFC. Meanwhile, areas like the BG, which primarily utilize dopamine reuptake, are not known to engage in persistent activity via excitatory feedback, not to mention that the striatum is composed of mostly GABAergic neurons (~95%) that are incapable of excitatory feedback.

What might occur within persistent activity circuits if HCY reaches pathological levels? We suggest above that low-level HCY accumulation after dopamine release may be a required feature of neural circuits that mediate working memory to reduce NMDAR desensitization. NMDAR desensitization, however, may be a built in way to prevent a sensitive neural architecture like recurrent excitatory collaterals from runaway feedback excitation. If HCY levels become pathologically high, NMDAR desensitization will be fully prevented, which may sustain persistent activity related to a correct answer past the current trial. In this case, previous answers could potentially “bleed” into future trials, which would result in perseverative answers. Moreover, patients will respond confidently because there is an actual false memory trace present instead of the absence of a memory trace. This is the exact phenotype of a schizophrenia patient when performing a spatial working memory task: perseverative answers from previously correct trials that are provided with confidence. Perseveration on previously correct rules in the WCST could also come about in this manner.

## Integration With Previous Theory and Future Directions

### General Schizophrenia Theory

The NMDAR hypofunction theory of schizophrenia has been a prominent hypothesis of the disease and is based on the fact that NMDAR antagonists like PCP and ketamine induce disease symptoms in healthy individuals (for review see Javitt, 2010). HCY, as shown above, has the reverse effect of PCP (i.e., it enhances current flow through NMDARs). We suggest that repeated current enhancement of NMDARs via HCY could induce NMDAR hypofunction via the homeostatic receptor internalization that occurs with prolonged excessive activity (as observed by Watt et al., 2000). This idea coalesces well with the fact that obligatory NR1 subunits have been found to be decreased by ~40% while NR1 mRNA is 60% decreased in the postmortem PFC of schizophrenia patients (for meta-analysis see Catts et al., 2016). Future experiments could address whether chronic HCY elevation in the blood is correlated with decreases in NMDA subunit expression over development. Further developmental effects could be induced by HCY given the critical role of NMDARs in LTP and LTD (Christie et al., 2005, 2009). Indeed, chronic dysregulated synaptic plasticity has been proposed as a mechanism for schizophrenia pathology (e.g., Sekar et al., 2016). Plasticity changes due to long-term NMDAR current enhancement by HCY must be disentangled from its acute effects on persistent activity in future studies.

### Working Memory-Specific Theory

Numerous studies have postulated a direct role for dysregulated dopamine signaling through the D1 receptor in working memory deficits (e.g., Goldman-Rakic, 1996, 1999). D1 binding drugs affect both working memory performance (Sawaguchi and Goldman-Rakic, 1994) and prefrontal persistent activity (Williams and Goldman-Rakic, 1995; Sawaguchi, 2001). Moreover, slice electrophysiology in the rodent PFC supports a role for D1 receptors in altering conductances that are required for proper working memory function (Seamans et al., 2001a,b). Based on these electrophysiological results, models of D1-modulated persistent activity have recapitulated many of the D1-related results observed in monkeys (Durstewitz and Seamans, 2002). We therefore believe that the dopamine system itself is likely critical for proper working memory function; our theory only seeks to augment current hypothesis by providing a second means of dopamine functionality during working memory. In fact, we believe that a main role for dopamine signaling in working memory supports our hypothesis because dopamine breakdown and dopamine receptor activation should be generally correlated in the PFC or SC. Our theory could further serve to supplement previous investigations into how COMT genotypes in humans affect working memory in normal populations and in schizophrenia (e.g., Meyer-Lindenberg and Weinberger, 2006; Durstewitz and Seamans, 2008). This will be addressed in more detail below. Finally, we believe our theory serves as a link between the aforesaid dopamine related results in monkeys and the similar phenotypes observed in monkeys dosed with NMDAR interacting drugs (Wang et al., 2013).

We propose two lines of research that could specifically address the working memory ideas put forth in this article. Starting with human genetics, there are multiple common polymorphisms in the genes that both produce (COMT 157Val/Met) and metabolize (MTHFR TT/CC) HCY. The Val COMT polymorphism produces an enzyme that is ~40% more active in metabolizing dopamine than the Met polymorphism (Chen et al., 2004). Similarly, the TT MTHFR polymorphism metabolizes HCY at a significantly lower rate than CC (Tunbridge et al., 2008). If an individual possesses both hyperactive COMT and hypoactive MTHFR, their HCY levels are significantly raised compared to other COMT/MTHFR genotypes (Tunbridge et al., 2008). Moreover, the polymorphisms of COMT and MTHFR that confer heightened HCY are both linked to schizophrenia susceptibility, with the slow HCY metabolizing TT MTHFR conferring a 36% enhanced risk of schizophrenia (Muntjewerff et al., 2006).

Interestingly, COMT and MTHFR polymorphisms that enhance HCY levels combine to produce working memory deficits in schizophrenia patients (Roffman et al., 2008). However, the working memory task given to the patients is not the same task that is typically associated with schizophrenia. We therefore suggest systematically measuring human performance on a well-established spatial working memory paradigm described in Figure 5 and used widely by neuroscientists, while genotyping for COMT and MTHFR variants. An analysis that examines these and other polymorphisms, levels of HCY, working memory performance, and disease state of the individual would go a long way towards uncovering not just whether HCY is linked to working memory deficits, but whether these deficits co-segregate with disease severity. We hypothesize that there is likely a set-point of HCY where excitatory feedback desensitization during WM is ideal. This level would prevent perseverative errors due to previous answers while slowing desensitization enough so that a modicum of NMDAR current can properly mediate seconds-long feedback. Therefore, any set of polymorphisms that either overly produce or overly restrict HCY should be detrimental to working memory. This is likely also the case for gene products that create or metabolize other desensitization altering molecules like glycine or d-serine (e.g., G72, DAAO). It would be especially interesting to study the recently created “humanized” mouse lines that are homozygous for the human Val or Met allele of COMT. Mice homozygous for the Val allele, which in humans confers heightened HCY (Tunbridge et al., 2008), show poor working memory relative to their homozygous Met littermates (Risbrough et al., 2014).

Second, a systems neuroscience approach should be taken to examine the role of HCY in working memory maintenance. Working memory paradigms should be utilized that resemble the tasks schizophrenia patients fail using model systems that are more tractable than humans or monkeys (e.g., Felsen and Mainen, 2012; Kopec et al., 2015). These model systems should allow the simultaneous recording or imaging of whole populations of neurons participating in persistent activity during working memory. To this end, larval zebrafish offer a good model system for this type of whole-microcircuit analysis considering their amenability to whole-brain calcium imaging and *in vivo* patch clamping (Ahrens et al., 2012; Grama and Engert, 2012).

It is already suspected that dopamine release is critical to WM maintenance due to DA neuron activity patterns and the effects of dopamine antagonists and agonists on working memory performance. However, fluorescent probes or electrophysiological methods indicating the presence of DA and HCY in persistently active populations haven’t been employed in the context of working memory (i.e., amperometry, two-photon imaging; Sarkar et al., 2014). It is critical for this theory to know that HCY is actually present and modulates NMDAR function in the immediate aftermath of dopamine release in recurrently active microcircuits. Probes that fluoresce when binding HCY have been developed (Zhang et al., 2014) but have never been tested in neurons. HCY uncaging has become possible with the development of UV-activatable designer amino acid molecules (Uprety et al., 2014). The effect of observed and induced HCY on microcircuits engaging in persistent activity should be assayed using detailed electrophysiology studies aimed at recording HCY’s effect on synaptic currents mediating persistent activity. This would go a long way towards linking our results from direct agonist-application experiments to the more realistic synaptic currents that are assumed to be desensitizing in our model. Other dopamine breakdown products (e.g., 3-MT, HVA) could also be examined for effects in this manner. Further pursuing this line of research could eventually link systems neuroscience to the clinical findings uncovered above and provide a clearer picture into the role of dopamine breakdown in schizophrenia. More broadly, the systems neuroscience approach could explain why there is a continuum of dopamine reuptake/breakdown in the brain and how each brain area uses dopamine breakdown products for its purposes.

## Author Contributions

MC-P supervised all graduate work by AB that lead to the development of the presented theory. AB conceived the theory and drafted the manuscript in consultation with MC-P. AB and MC-P edited and revised the manuscript and approved the final version.

## Conflict of Interest Statement

The authors declare that the research was conducted in the absence of any commercial or financial relationships that could be construed as a potential conflict of interest.
